# The biological dimension of endodontic irrigants - beyond antimicrobial efficacy

**DOI:** 10.21142/2523-2754-1404-2026-309

**Published:** 2026-10-01

**Authors:** Hernán Coaguila-Llerena

**Affiliations:** Section of Endodontics, Faculty of Stomatology, Cayetano Heredia Peruvian University (UPCH). Lima, Peru. ehernanco@gmail.com Section of Endodontics Faculty of Stomatology Cayetano Heredia Peruvian University (UPCH) Lima Peru ehernanco@gmail.com

Traditionally, endodontic irrigants have been almost exclusively judged for what they kill or solve, e.g., bacteria, biofilm, or residual pulp tissue. However, the same chemical properties that make sodium hypochlorite (NaOCl), chlorhexidine (CHX), and ethylenediaminetetraacetic acid (EDTA) effective disinfectants, alone or in association, also determine how these solutions interact with periapical tissues, dentin integrity, dentin-embedded growth factors, and the stem cells. These factors are particularly relevant not only for conventional root canal treatment but also to regenerative endodontics, wherein the irrigant must eliminate infection while preserving the viability and function of cells involved in tissue repair. ^1^ Thus, when selecting endodontic irrigants, biocompatibility can no longer be considered a secondary issue; rather, it must be weighed alongside antimicrobial efficacy.

The biological effects of these solutions illustrate this complexity. For example, accidental extrusion of NaOCl has been extensively reported in the literature and can cause tissue injury related to its inherent cytotoxicity. Such accidents may result in an unpleasant experience for both patients and clinicians. ^2^ On the regenerative side, EDTA has been shown to favor dental pulp stem cell adhesion, migration, and odontoblastic differentiation on conditioned dentin ^3^. A different scenario is observed with NaOCl and CHX, which promote lower biocompatibility towards dental-derived mesenchymal stem cells. ^4^ These findings highlight that different endodontic irrigants produce divergent biological responses, reinforcing that biological outcome, not merely antimicrobial spectrum, should guide irrigant selection in regenerative endodontics. ^5^

The challenge for contemporary irrigation is therefore to achieve effective disinfection without unnecessary collateral biological effects. Evidence shows that no single irrigant simultaneously provides optimal antimicrobial activity, tissue dissolution, chelation, biocompatibility, and preservation of dentinal properties. This should foster a paradigm shift-moving from asking which irrigant is the "best" to determining which irrigation strategy is biologically appropriate for the specific treatment objective. NaOCl remains fundamental for its tissue-dissolving and antibacterial action, but its biological cost demands vigilance. EDTA and CHX, in turn, cannot be selected on convention alone; their differential effects on stem cell viability and growth factor exposure carry direct consequences for regenerative success. As postgraduate education increasingly incorporates regenerative endodontics into its curriculum, the biological properties of irrigants deserve the same scrutiny and clinical respect traditionally reserved for their antimicrobial credentials.
